# Nucleus Accumbens Functional Connectivity with the Frontoparietal Network Predicts Subsequent Change in Body Mass Index for American Children

**DOI:** 10.3390/brainsci10100703

**Published:** 2020-10-03

**Authors:** Shervin Assari, Shanika Boyce, Mohsen Bazargan

**Affiliations:** 1 Department of Family Medicine, Charles Drew University, Los Angeles, CA 90059, USA; mohsenbazargan@cdrewu.edu; 2 Department of Urban Public Health, Charles Drew University, Los Angeles, CA 90059, USA; 3 Department of Pediatrics, Charles Drew University, Los Angeles, CA 90059, USA; shanikaboyce@cdrewu.edu; 4 Department of Family Medicine, University of California Los Angeles (UCLA), Los Angeles, CA 90095, USA

**Keywords:** children, obesity, nucleus accumbens, body mass index, frontoparietal

## Abstract

**Background:** Nucleus accumbens (NAc) is a brain structure with a well-established role in the brain reward processing system. Altered function of the NAc is shown to have a role in the development of food addiction and obesity. However, less is known about sex differences in the role of NAc function as a predictor of children’s change in body mass index (BMI) over time. Aim: We used the Adolescent Brain Cognitive Development data (version 2.01) to investigate sex differences in the predictive role of the NAc functional connectivity with the frontoparietal network on children’s BMI change over a one-year follow-up period. **Methods:** This 1-year longitudinal study successfully followed 3784 9–10-year-old children. Regression models were used to analyze the data. The predictor variable was NAc functional connectivity with the frontoparietal network measured using resting-state functional magnetic resonance imaging (fMRI). The primary outcome was BMI at the end of the 1-year follow up. Covariates included race, ethnicity, age, socioeconomic factors, and baseline BMI. Sex was the effect modifier. **Results:** NAc functional connectivity with the frontoparietal network was predictive of BMI changes over time. This association remained significant above and beyond all covariates. The above association, however, was only significant in female, not male children. **Conclusion:** The epidemiological observation that NAc functional connectivity is associated with BMI changes in children is an extension of well-controlled laboratory studies that have established the role of the NAc in the brain reward processing. More research is needed on sex differences in the brain regions that contribute to childhood obesity.

## 1. Introduction

Nucleus accumbens (NAc), a central region in the basal ganglia, has a significant role in Pavlovian learning [1,2,3,4,5,6]. NAc is involved in regulation of incentive salience, pleasure-seeking, reward dependence, and positive reinforcement [7,8,9,10,11,12]. As such, altered NAc function is central to food, tobacco, alcohol, and drug-seeking behaviors [13,14,15,16,17]. By regulating the response to food cues, the NAc also plays a unique role in pathogenesis of food addiction, eating disorders, [1,18,19,20], obesity, and high body mass index (BMI) [21].

The NAc mediates cue-triggered reward-seeking behaviors [9,18,22,23,24]. In animal models [25] and humans [26,27,28,29,30,31], the obesity-prone differ from the obesity-resistant in NAc activity. Due to its central role in incentive motivation, the NAc is involved in the expression of behaviors that contribute to the development of obesity [25]. Food cues are shown to elicit a robust NAc dopamine response at the time of hunger [21]. Thus, dopamine release in the NAc alters how an individual seeks food [17,29,32,33,34]. Activity in the NAc is particularly enhanced in obesity-susceptible people [25]. Although we know that NAc has a prominent role in the development of obesity, most of the existing evidence is limited to animal studies, small-sized human studies in a highly controlled laboratory settings, or cross-sectional studies [26,27,28,29,30,31]. As such, we know little about the role of the NAc in shaping future risk of obesity in the general population.

The NAc, however, does not perform its functions in a vacuum as it receives projections from a plethora of brain regions and networks. Among the various brain networks that work with the basal ganglia, striatum, and NAc is the frontoparietal network [35]. Such a connectivity is linked to food preference [36], obesity [37,38,39,40,41], and eating disorders [42,43]. Via frontoparietal-accumbal connectivity, frontoparietal network and NAc joint function is involved in motivated behavior, food seeking, emotion regulation, and obesity [37,38,39,40,41]. It has also shown that NAc functional connectivity with frontoparietal-accumbal may have some role in dopaminergic and reward systems of the brain [44]. As a result, the functional connectivity of the frontoparietal network and the NAc may have a role as a predictor factor for the BMI at baseline and change over time [37,38,39,40,41].

### Aims

Although NAc function is a well-established predictor of food-seeking behaviors [21], less is known about the role of connectivity between the NAc and frontoparietal network on obesity [37,38,39,40,41]. While we know that NAc function is different in obesity-prone individuals than others [26,27,28,29,30,31], there is a need for longitudinal epidemiological studies on the NAc-frontoparietal connectivity as a predictor of obesity risk at the population level [21]. This is important because individuals are also under the influence of their social context and environment, which can also predict BMI changes. In addition, even less is known about sex differences in the role that functional connectivity between the NAc and frontoparietal network has in predicting children’s BMI change over time. To respond to this gap in the literature, we conducted an epidemiological study of 9–10-year-old children in the U.S. to test if NAc functional connectivity with the frontoparietal network at baseline is associated with future BMI change over a one-year period. Our second aim was to test sex differences in the above association.

## 2. Materials and Methods

### 2.1. Design and Settings

A secondary analysis was performed with a cross-sectional design. We used data from the Adolescent Brain Cognitive Development (ABCD) study [45,46,47,48,49]. ABCD, a landmark study of brain development from childhood to emerging adults, is unique in the United States. Although details of the ABCD methods, measures, design, sample, and sampling are described elsewhere [45,50], we briefly review them.

### 2.2. Participants and Sampling

In the ABCD, we only included children who were between the ages of 9 and 10 years. The ABCD children were enrolled from multiple cities across the United States. Overall, children were recruited to the ABCD study from 21 sites. The primary strategy for sampling in the ABCD study was the school systems [51]. In the current analysis, 3784 participants entered. Our analysis’ eligibility for inclusion was 9–10-year-old non-twin participants who had data on the baseline and follow up BMI at the end of the first year. Participants could be from any race or ethnicity.

### 2.3. Neuroimaging Data Have Been Processed

We used neuroimaging data which were already processed. The ABCD Magnetic Resonance Imaging (MRI) data include functional and structural MRI. Functional MRI (fMRI) includes resting-state and task-based fMRI. Resting-state fMRI was used for this analysis. Beta correlation between the frontoparietal network and the automatic subcortical segmentation (ASEG) region of interest (ROI) right-accumbens area was calculated. Tesla 1 (T1) and T2 weighted fMRI and sMRI images were taken using a 3 tesla (T) Siemens Prisma (Erlangen, Germany), General Electric 750 (Chicago, IL, USA), and Phillips (Amsterdam, The Netherlands) multi-channel coiled scanners, all capable of multiband echo-planar imaging (EPI) acquisitions. A localizer was implemented at the beginning of each scan, followed by a T1 weighted fMRI image acquisition. Functional T2 weighted scans were then acquired at rest and then throughout three psychological tasks. A large-scale multimodal data acquisition allowed the ABCD study to collect an unprecedented set of images on a large number of adolescents that were selected and enrolled via 21 data acquisition sites across various U.S. states. As mentioned above, both ABCD structural and functional MRI data are processed and the tabulated regions of interest (ROI) data were downloaded from the National Institute of Health (NIH) Data Archive (NDA). ABCD imaging data were limited to resting-state fMRI. More information on the imaging protocols are available here [52,53,54].

### 2.4. Study Variables

#### 2.4.1. Outcome

*Body Mass Index (BMI).* The children’s BMI at baseline and at the end of the 1-year follow-up was calculated based on participants’ measured height and weight. Height was measured three times in inches. The weight of the child was measured up to three times in pounds. BMI was treated as a continuous measure. Although some research has used percentile BMI related to age and sex norms, we used raw BMI because the analyses broke the sample by sex and the age range of the participants was very narrow (only 9- and 10-year-old children). Other research has also used BMI rather than percentile BMI related to age and sex norm.

#### 2.4.2. Independent Variable

*NAc.* Using resting-state fMRI, NAc was defined as the average correlation between the frontoparietal network and ASEG ROI right-accumbens area. This is functional connectivity between the frontoparietal network and the NAc. The resting-state fMRI was measured at baseline at the same time that socioeconomic status (SES) indicators and baseline BMI were measured.

#### 2.4.3. Confounders

*Race.* Race, a self-identified variable, was two binary variables: Blacks, other races, and Whites (reference category).

*Ethnicity.* Ethnicity, a self-identified variable, was 1 for Hispanics and 0 for non-Hispanics (reference category).

*Sex.* A dichotomous variable, sex was coded as below: males = 1, females = 0.

*Age.* Age (months), calculated as the difference between birth and the time of enrollment to the study, measured in months, was reported by parents.

*Parent education (y).* Parents reported their years of schooling. This variable was operationalized as a continuous (interval) variable ranging from 0 for no formal education to 21 for doctoral degrees.

*Parent employment.* Parents reported their employment. This variable was operationalized as a categorical variable with 0 for non-employed and 1 for employed.

*Family income.* Family income was a 1–10 interval measure where a higher score indicated higher income. The total combined family income in the past 12 months was asked. Responses were 1 = less than USD 5000; 2 = USD 5000; 3 = USD 12,000; 4 = USD 16,000; 5 = USD 25,000; 6 = USD 35,000; 7 = USD 50,000; 8 = USD 75,000; 9 = USD 100,000; 10 = USD 200,000.

### 2.5. Main Data Analysis

The statistical package, SPSS 22.0 (IBM, Armonk, NY, USA), was applied for data analysis. Mean (standard deviation; SD) and frequency (relative frequency; %) was used to describe all the study variables. We also used the Pearson correlation test for bivariate analysis of the associations between the study variables. For multivariable modeling, we ran four regression models. To do so, first we tested the assumptions that are required for running a regression model. BMI time 1 and time 2 had a near to normal distribution. There is no collinearity between the study variables. In our model, the NAc functional connectivity with the frontoparietal network was used as the independent variable (predictor), baseline BMI and demographic and SES indicators as the covariates and follow-up BMI as the outcome. Our 1st two models were performed in the pooled sample. Our 1st model was without (*Model 1*), and our 2nd model was with (*Model 2*) an interaction term between sex and NAc functional connectivity with the frontoparietal network. Our next models were run for females (*Model 3*) and males (*Model 4*). Models were identical for right and left NAc. All models were statistically significant at a 0.001 and explained more than 20% of the variance of the outcome, mainly because the outcome and independent variable (predictor) had a strong correlation (baseline and time 2 BMI). This information is added to the statistical analysis section of the paper. We did not adjust the BMIs based on the age and sex of the participants because the BMI values of each individual were being compared at two time points (baseline and follow up BMI). As every individual is compared to his/herself, there is no need to adjust based on the distribution of BMI in the population. There are several studies that have used the same approach and have not adjusted based on norms. Unstandardized coefficient (b), standard error (SE), 95% confidence interval (95% CI), and *p*-value were reported for our model. A *p*-value equal or less 0.05 was significant.

### 2.6. Sensitivity Analysis

In our main analysis, the outcome was BMI time 2, while BMI time 1 and all covariates were controlled. We ran a mixed effects model with BMI time 1 as the outcome. The argument behind this approach if the result of our main analysis with BMI time 2 is robust is that we should test the same interaction in our replication model, despite the sample size and the temporal aspects of the measures having changed. As our findings show, our sensitivity analysis replicated the same interaction between sex and NAc functional connectivity with the frontoparietal network on BMI (Appendix A Tables and Figures).

### 2.7. Ethics

The ABCD study protocol received Institutional Review Board (IRB) approval from several institutions, including but not limited to the University of California, San Diego (UCSD). All participating children provided assent. All participating parents signed informed consent [50]. As we only performed a secondary analysis of fully de-identified data, our study was non-human subject research. Thus, our analysis and report did not require an IRB review (exempt from a full IRB review).

## 3. Results

### 3.1. Descriptives

A total number of 3784, 9–10-year-old children were analyzed. Participants were 1953 male and 1831 female children. Table 1 presents a summary of the descriptive statistics for the children overall and by sex.

### 3.2. Bivariate Correlations

Table 2 shows a summary of bivariate correlations. Functional connectivity between the frontoparietal network and the NAc was inversely correlated with follow-up BMI. The bivariate correlation between right and left functional connectivity between NAc and frontoparietal network was almost zero. Although baseline BMI was not associated with right and left functional connectivity between the NAc and frontoparietal network, follow-up BMI was correlated with right and left functional connectivity between the NAc and frontoparietal network. Baseline and follow-up BMI were positively correlated; however, this correlation was not very strong. From various SES indicators, parental marital status and parental education were correlated with right but not left functional connectivity between the NAc and frontoparietal network.

### 3.3. Multivariate Analysis

Table 3 shows a summary of the results of two regression models in the pooled sample. These models show a significant and negative effect of NAc functional connectivity with the frontoparietal network on follow-up BMI in children. This means a higher beta coefficient that reflected higher NAc functional connectivity with the frontoparietal network was associated with lower BMI gain over time. Results were identical for right and left NAc.

### 3.4. Multivariate Analysis

Table 4 shows a summary of the results of two regression models in female and male children. These models show a significant and negative effect of NAc functional connectivity with the frontoparietal network on follow-up BMI in females (b = −23.93; *p* = 0.012 for right and b = −32.31; *p* = 0.000 for left NAc functional connectivity with the frontoparietal network) but not males (b = −0.41; *p* = 0.227 for right and b = 0.15; *p* = 0.621 for left NAc functional connectivity with the frontoparietal network). Results were identical for right and left NAc. BMI baseline was also positively and significantly associated with time 2 BMI, which is reflective of autoregressive correlation (b = 0.90 for females and 0.98 for males).

### 3.5. Sensitivity Analysis

As the appendix shows, we ran a full series of sensitivity analyses to test if we could successfully replicate the results of our main analysis. As shown above, our main analysis shows that the effect of NAc functional connectivity with the frontoparietal network on BMI time 2, while covariates and BMI time 1 was controlled. We ran a mixed effects model with BMI time 1 as the outcome. The argument behind this approach is if the result of our main analysis with BMI time 2 is robust, then we should test the same interaction in our replication model, despite the sample size and the temporal aspects of the measures having changed. As our findings show, our sensitivity analysis replicated the same interaction between sex and NAc functional connectivity with the frontoparietal network on BMI (Appendix A Tables and Figures). That means that not only does the effect of NAc functional connectivity with the frontoparietal network on future BMI depend on sex, but the cross-sectional association between NAc functional connectivity with the frontoparietal network and BMI also statistically differs between male and female children.

## 4. Discussion

Our first main finding suggested that the NAc functional connectivity with the frontoparietal network was associated with 1-year BMI among 9–10-year-old American children. According to our second main finding, this predictive role was true for female children, but not male children. We also replicated these main results by cross-sectional association between NAc functional connectivity with the frontoparietal network and BMI, which also differed by sex. Another source of confidence in our results was that the findings were identical for right and left NAc.

Our first main finding is supported by the lab-based observations that the basal ganglia (striatum and NAc) and frontoparietal network function together [35], and that their functional connectivity is also linked to food preference [36], obesity [37,38,39,40,41], and eating disorders [42,43]. An extensive body of laboratory research has shown that the NAc shell receives dopaminergic inputs from various structures due to various sensory inputs including those related to food [26,55,56,57,58,59,60]. The NAc is involved in the regulation of feeding, eating, and food seeking behaviors [1,7,8,20,24,55,61]. Gamma-aminobutyric acid-_A_ (GABA_A_) receptors (a receptor for the GABA hormone released by the brain to regulate dopamine levels in its reward pathways) in the NAc shell mediate hyperphagia, overeating, and associated weight gain [62]. NAc controls appetite as well [63]. Motivational responses to food are mediated in part by NAc α-amino-3-hydroxy-5-methyl-4-isoxazolepropionic acid (AMPA) receptor transmission [64]. NAc medium spiny neurons (MSNs) are hyper-responsive in obesity-prone individuals [34]. The NAc’s interaction with predisposition factors and obesogenic environment may alter neurobehavioral plasticity in the NAc, which promotes weight gain and reduces weight loss in obesity-susceptible animals and people [34]. Recent work shows that cue-triggered motivation is enhanced in obesity-susceptible individuals after consuming “junk-food” [64]. Chronic and repeated exposure to food cues and specific diets may result in changes within the NAc, a part of the mesolimbic pathway that regulates food seeking behaviors [65]. The NAc’s interaction with predisposition factors and an obesogenic environment may alter neurobehavioral plasticity in the NAc which may promote weight gain and reduce weight loss among obesity-susceptible animals and people [34].

Our second main finding, the predictive role of the frontoparietal network and NAc functional connectivity on BMI time 2 of female but not male children is very novel. An extensive body of research has previously shown that sex alters predictors of obesity and BMI change over time [66,67,68,69,70]. Socioeconomic status, mood regulation, and stress all differently correlate with BMI of males and females [66,67,68,69,70]. In many studies, obesity and BMI change have shown more associations in females than males [66,67,68,69,70]. This might be because females are more vulnerable to the risk factors of obesity, given their environmental situation, societal status, and coping mechanisms [66,68,69,70,71,72,73,74,75,76], or simply because obesity has a larger variance in females so our existing models can better explain BMI variation in females than males.

In our main analysis, we had follow-up BMI as the outcome and baseline BMI as a covariate. This is one of the two main approaches to analyze the repeated measure outcome only with two observations. Maria Glymour and others have discussed how controlling for baseline status of an outcome alters the interpretation of the results. We decided to control for baseline BMI when the outcome was time 2 BMI based on the causal diagram [77]. Still, to replicate the results in the same study, we ran a series of sensitivity analysis with BMI time 1 as the outcome. The interaction term between sex and NAc functional connectivity with the frontoparietal network on baseline BMI suggested that our results are robust.

### 4.1. Implications

Function of the NAc is believed to be modifiable through neuro-modulation [78]. NAc function has been modulated in both research and clinical settings [20,79,80,81,82]. Smoking cessation and weight loss programs, for example, have used modalities that leverage NAc modification [78]. Some programs that have stimulated the NAc have generated promising results in reducing food craving, as well as treatment of obesity-induced food addiction and other cue-related disorders [78]. Deep brain stimulation of the NAc is also a technique that can be implemented in therapies [20,55,79,80,81,82,83,84,85].

### 4.2. Limitations

All studies, particularly those who are secondary analysis of some existing data, are limited in their design, methods, and measurements. NAc activity and high BMI may have bidirectional effects. However, we used a longitudinal design and established the longitudinal effect of baseline NAc function on children’s BMI change over time. We only had a few confounders, and we did not have data on diet, food cues, obesogenic environment, mental health, emotion regulation, and food addiction. Finally, we should emphasize that in this study, our outcome was not a dichotomous variable of obesity but the continuous measure of BMI. As such, the result should not be interpreted as NAc function predicts obesity, but BMI value at time 2 (while BMI value at time 1 is controlled). That means, regardless of passing or not passing the threshold that we use to define obesity or overweight, NAc function predicts who develops a higher BMI over time.

### 4.3. Future Research

As the duration of follow up in our study was short, long-term longitudinal data are needed. Imaging studies on longitudinal association between the NAc, and behavioral development are needed. We only described a link between the NAc and BMI change. Future research should study the mechanism of the effects of the NAc on BMI change. Future work is needed on various social, behaviors, and brain mechanisms that can explain how baseline NAc function predicts future obesity, and how these paths may differ for various groups of American children. As the NAc is not only responsive to food but also other categories of rewards such as alcohol [65] and sex [86,87], future research should replicate this result for other cue-induced behaviors. Research should also investigate how food environment and past behaviors modulate the food cue-induced behaviors that regulate eating through a change in the NAc activity. The same can be relevant to tobacco, alcohol, illicit drugs, and sex. Changes in the NAc may regulate, promote, or inhibit tobacco, food, alcohol, and drug seeking behaviors [88,89,90,91,92]. At a public health level, knowledge regarding the role of the NAc on BMI and obesity may help us undo social, economic, and environmental inequalities in childhood obesity. It is important to study how social distribution of food cues generate inequalities and disparities in the burden of obesity in children. We need to study environmental risk factors that impact NAc function across populations of children. We need to know the societal and behavioral conditions that may alter the function of the NAc in children.

## 5. Conclusions

In this longitudinal epidemiological study, the NAc functional connectivity with the frontoparietal network was predictive of future BMI increase among female but not male American children. The NAc, the frontoparietal network, and their functional connectivity may be a part of the brain circuits involved in the development of obesity among female children.

## Figures and Tables

**Table 1:** Data overall (*n* = 3784).

	All		Female		Male	
	**n**	**%**	**n**	**%**	**n**	**%**
**Sex**						
Female	1831	48.4	1831	100.0	-	-
Male	1953	51.6	-	-	1953	100.0
**Age**						
9	1811	47.9	902	49.3	909	46.7
10	1966	52.1	927	50.7	1039	53.3
**Child ethnicity**						
Non-Hispanic	3015	79.7	1464	80.0	1551	79.4
Hispanic	769	20.3	367	20.0	402	20.6
**Child race**						
White	2914	77.0	1382	75.5	1532	78.4
Other	334	8.8	176	9.6	158	8.1
Black	536	14.2	273	14.9	263	13.5
**Parental employment**						
No	1153	30.5	545	29.8	608	31.1
Yes	2631	69.5	1286	70.2	1345	68.9
**Parents married**						
No	1049	27.7	510	27.9	539	27.6
Yes	2735	72.3	1321	72.1	1414	72.4
	**Mean**	**SD**	**Mean**	**SD**	**Mean**	**SD**
Parent education (y)	16.89	2.63	16.96	2.59	16.82	2.66
Family income (1–10)	7.51	2.17	7.56	2.12	7.46	2.22
BMI (baseline)	18.53	3.93	18.59	4.03	18.48	3.84
BMI (1 y) *	20.10	3.7.	20.88	5.37	19.37	4.43
Right NAc functional connectivity with the frontoparietal network	−0.01	0.15	−0.01	0.14	−0.01	0.16
Left NAc functional connectivity with the frontoparietal network	−0.05	0.17	−0.06	0.16	−0.05	0.17

Nucleus accumbens (NAc): average correlation between frontoparietal network and right- and left-accumbens area; SD = standard deviation, BMI = body mass index. * *p* < 0.05 for a comparison of males and females.

**Table 2:** Correlations in the total sample.

	1	2	3	4	5	6	7	8	9	10	11	12	13
1. Sex (Male)	1	0.03	−0.03	−0.02	0.01	0.00	−0.02	−0.03	−0.02	−0.01	−0.02	−0.02	0.03 *
2. Age (10 y)		1	0.01	0.01	−0.01	0.00	0.02	0.04 *	0.05 **	0.09 **	−0.01	0.00	−0.01
3. Race (Black)			1	−0.13 **	0.27 **	−0.08 **	−0.04 *	−0.17 **	−0.15 **	0.05 **	0.00	−0.03	−0.02
4. Race (Other)				1	−0.09 **	−0.29 **	−0.01	−0.13 **	−0.28 **	0.16 **	0.01	−0.04 *	0.04 **
5. Ethnicity (Hispanic)					1	−0.15 **	−0.07 **	−0.37 **	−0.31 **	0.18 **	0.01	−0.01	0.00
6. Parents married						1	0.02	0.29 **	0.49 **	−0.15 **	−0.05 **	0.05 **	−0.03
7. Parents employed							1	0.23 **	0.23 **	−0.01	0.01	0.02	0.03
8. Parent education (y)								1	0.60 **	−0.18 **	−0.01	0.04 *	−0.02
9. Family Income									1	−0.20 **	−0.01	0.02	0.00
10. BMI (baseline)										1	0.10 **	−0.03	0.00
11. BMI (1 y)											1	−0.04 **	−0.06 **
12. Right NAc												1	0.00
13. Left NAc													1

NAc: average correlation between the frontoparietal network and ASEG ROI right- and left-accumbens area; BMI = body mass index; * *p* < 0.05; ** *p* < 0.01.

**Table 3:** Overall regression models.

	b	SE	95% CI		t	*p*	b	SE	95% CI		t	*p*
			Right						Left			
**Model 1**												
Sex (Male)	−1.45	1.32	−4.05	1.14	−1.10	0.272	−1.20	1.32	−3.79	1.38	−0.91	0.362
Age (10 y)	−1.32	1.33	−3.93	1.29	−0.99	0.320	−1.42	1.33	−4.01	1.18	−1.07	0.285
Race (Other)	−0.66	2.54	−5.65	4.33	−0.26	0.796	−0.66	2.54	−5.64	4.32	−0.26	0.796
Race (Black)	−1.33	2.11	−5.46	2.81	−0.63	0.529	−0.73	2.10	−4.85	3.39	−0.35	0.729
Ethnicity (Hispanic)	−0.35	1.89	−4.06	3.36	−0.19	0.853	−0.30	1.89	−4.00	3.40	−0.16	0.874
Married household	−3.91	1.75	−7.34	−0.47	−2.23	0.026	−4.21	1.75	−7.64	−0.79	−2.41	0.016
Parents employed	0.52	1.52	−2.47	3.50	0.34	0.734	0.62	1.52	−2.36	3.59	0.41	0.684
Parent education years (1–21)	0.09	0.34	−0.58	0.76	0.27	0.787	0.05	0.34	−0.62	0.72	0.14	0.886
Family income	0.33	0.44	−0.53	1.18	0.74	0.456	0.39	0.44	−0.47	1.25	0.89	0.371
BMI baseline	0.93	0.18	0.58	1.27	5.26	< 0.001	0.92	0.18	0.58	1.27	5.25	< 0.001
Accumbens area	−11.43	4.50	−20.25	−2.61	−2.54	0.011	−14.51	4.00	−22.35	−6.66	−3.63	< 0.001
**Model 2**												
Sex (Male)	−1.21	1.32	−3.81	1.38	−0.92	0.360	0.43	1.38	−2.28	3.13	0.31	0.757
Age (10 y)	−1.41	1.33	−4.01	1.20	−1.06	0.289	−1.40	1.32	−4.00	1.19	−1.06	0.289
Race (Other)	−0.83	2.54	−5.82	4.15	−0.33	0.743	−0.68	2.53	−5.65	4.29	−0.27	0.788
Race (Black)	−1.15	2.11	−5.29	2.98	−0.55	0.584	−0.87	2.10	−4.99	3.24	−0.42	0.677
Ethnicity (Hispanic)	−0.42	1.89	−4.13	3.29	−0.22	0.826	−0.54	1.89	−4.24	3.15	−0.29	0.773
Married household	−3.81	1.75	−7.25	−0.38	−2.18	0.030	−4.13	1.74	−7.55	−0.71	−2.37	0.018
Parents employed	0.50	1.52	−2.48	3.48	0.33	0.741	0.72	1.51	−2.25	3.69	0.48	0.634
Parent education years (1–21)	0.09	0.34	−0.58	0.76	0.27	0.789	0.04	0.34	−0.62	0.71	0.13	0.898
Family income	0.31	0.44	−0.55	1.17	0.71	0.477	0.37	0.44	−0.49	1.22	0.84	0.400
BMI baseline	0.93	0.18	0.58	1.27	5.25	< 0.001	0.93	0.18	0.58	1.27	5.29	< 0.001
Accumbens area	−24.14	6.58	−37.04	−11.23	−3.67	< 0.001	−31.90	5.94	−43.54	−20.26	−5.37	< 0.001
Sex (male) × Accumbens area	23.79	9.01	6.13	41.45	2.64	0.008	31.69	8.00	16.00	47.38	3.96	< 0.001

NAc: average correlation between the frontoparietal network and ASEG ROI right- or left-accumbens area (rsfmri_cor_ngd_fopa_scs_aarh or rsfmri_cor_ngd_fopa_scs_aalh); Outcome: BMI at the end of follow up; unstandardized regression coefficient: b; statistic: t; standard error: SE; confidence interval: CI; BMI = body mass index.

**Table 4:** Sex-specific regression models.

	b	SE	95% CI		t	*p*	B	SE	95% CI		t	*P*
			Right						Left			
**Model 3**												
Age (10 y)	−2.97	2.75	−8.37	2.43	−1.08	0.281	−2.98	2.74	−8.36	2.39	−1.09	0.276
Race (Other)	−1.39	5.04	−11.28	8.50	−0.28	0.783	−1.12	5.02	−10.97	8.73	−0.22	0.823
Race (Black)	−2.39	4.25	−10.72	5.93	−0.56	0.573	−1.90	4.23	−10.20	6.40	−0.45	0.654
Ethnicity (Hispanic)	−1.39	3.95	−9.14	6.35	−0.35	0.724	−1.72	3.94	−9.44	6.00	−0.44	0.662
Married household	−7.74	3.58	−14.78	−0.71	−2.16	0.031	−8.39	3.57	−15.38	−1.39	−2.35	0.019
Parents employed	0.96	3.16	−5.25	7.17	0.30	0.762	1.45	3.16	−4.75	7.64	0.46	0.647
Parent education years (1–21)	0.20	0.72	−1.21	1.62	0.28	0.779	0.10	0.72	−1.31	1.51	0.14	0.891
Family income	0.73	0.90	−1.03	2.49	0.82	0.415	0.84	0.89	−0.91	2.60	0.94	0.345
BMI baseline	0.90	0.36	0.20	1.59	2.51	0.012	0.90	0.36	0.20	1.60	2.53	0.012
Accumbens area	−23.93	9.48	−42.52	−5.33	−2.52	0.012	−32.31	8.55	−49.07	−15.55	−3.78	< 0.001
**Model 4**												
Age (10 y)	−0.06	0.10	−0.26	0.14	−0.54	0.586	−0.05	0.10	−0.25	0.15	−0.49	0.626
Race (Other)	−0.06	0.20	−0.46	0.34	−0.30	0.765	−0.06	0.20	−0.46	0.34	−0.29	0.771
Race (Black)	0.18	0.17	−0.15	0.50	1.07	0.284	0.19	0.17	−0.13	0.52	1.15	0.250
Ethnicity (Hispanic)	0.19	0.14	−0.09	0.47	1.32	0.189	0.18	0.14	−0.10	0.47	1.27	0.203
Married household	−0.07	0.14	−0.34	0.20	−0.50	0.614	−0.07	0.14	−0.34	0.19	−0.54	0.586
Parents employed	0.11	0.12	−0.11	0.34	0.98	0.325	0.10	0.12	−0.12	0.33	0.89	0.372
Parent education years (1–21)	−0.02	0.03	−0.07	0.03	−0.86	0.391	−0.02	0.03	−0.07	0.03	−0.85	0.396
Family income	−0.05	0.03	−0.11	0.02	−1.37	0.170	−0.05	0.03	−0.11	0.02	−1.41	0.159
BMI baseline	0.98	0.01	0.96	1.01	70.80	< 0.001	0.98	0.01	0.96	1.01	70.85	< 0.001
Accumbens area	−0.41	0.34	−1.08	0.26	−1.21	0.227	0.15	0.30	−0.44	0.73	0.49	0.621

NAc: average correlation between the frontoparietal network and right- or left-accumbens area (rsfmri_cor_ngd_fopa_scs_aarh or rsfmri_cor_ngd_fopa_scs_aalh); Outcome: BMI at the end of follow up; unstandardized regression coefficient: b; statistic: t; standard error: SE; confidence interval: CI.

**Figure A1:**
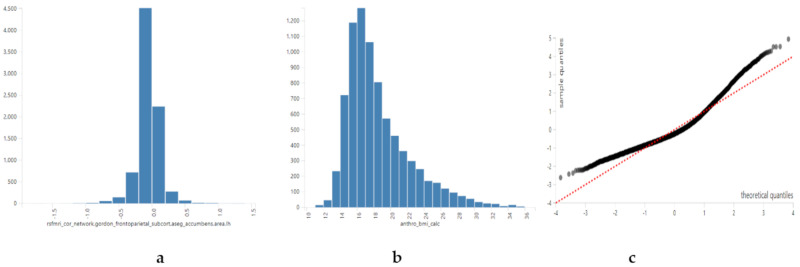
Assumptions for mixed-effects models for replication models. (**a**) histogram of the predictor (NAc functional connectivity with the frontoparietal network), (**b**) histogram of the outcome (baseline body mass index; BMI), (**c**) observed vs. theorized quartiles.

**Figure A2:**
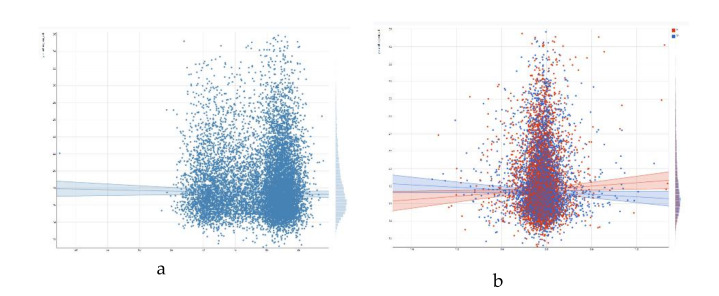
Regression coefficient of the effect of NAc functional connectivity with the frontoparietal network on BMI at baseline. (**a**) Overall association (blue), (**b**) association in females (red) and males (blue).

## Appendix A

**Table A1:** Formula for replication models.

anthro_bmi_calc ~ rsfmri_cor_network.gordon_frontoparietal_subcort.aseg_accumbens.area.lh + race.4level + sex + high.educ.bl + married.bl + age + household.income.bl + hisp + rsfmri_cor_network.gordon_frontoparietal_subcort.aseg_accumbens.area.lh * sex Random: ~(1|abcd_site/rel_family_id)

**Table A2:** Distribution of study variables based on sex.

	Level	All	F	M	*p*
N		10,184	3909	4168	
BMI time 1 (mean (SD))		18.65 (3.90)	18.62 (3.91)	18.41 (3.69)	0.013
NAc functional connectivity with the frontoparietal network (left) (mean (SD))		79.01 (5.06)	−0.06 (0.16)	−0.04 (0.18)	0.001
Race (%)	White	6802 (66.8)	2576 (65.9)	2859 (68.6)	0.051
	Black	1456 (14.3)	570 (14.6)	566 (13.6)	
	Asian	218 (2.1)	93 (2.4)	78 (1.9)	
	Other/Mixed	1708 (16.8)	670 (17.1)	665 (16.0)	
Sex (%)	Female	4860 (47.7)	3909 (100.0)	0 (0.0)	<0.001
	Male	5324 (52.3)	0 (0.0)	4168 (100.0)	
Parental Education (%)	<High School Diploma	369 (3.6)	144 (3.7)	117 (2.8)	0.142
	High School Diploma/ General Educational Development	837 (8.2)	304 (7.8)	339 (8.1)	
	Some College	2604 (25.6)	987 (25.2)	1110 (26.6)	
	Bachelor	2698 (26.5)	1061 (27.1)	1124 (27.0)	
	Postgraduate Degree	3676 (36.1)	1413 (36.1)	1478 (35.5)	
Family Married (%)	No	3082 (30.3)	1190 (30.4)	1195 (28.7)	0.085
	Yes	7102 (69.7)	2719 (69.6)	2973 (71.3)	
Age (mean (SD))		118.96 (7.47)	118.93 (7.47)	119.24 (7.53)	0.060
Household Income (%)	(<50K)	2900 (28.5)	1118 (28.6)	1157 (27.8)	0.508
	(≥50K and <100K)	2928 (28.8)	1145 (29.3)	1204 (28.9)	
	(≥100K)	4356 (42.8)	1646 (42.1)	1807 (43.4)	
Hispanic (%)	No	8259 (81.1)	3157 (80.8)	3390 (81.3)	0.531
	Yes	1925 (18.9)	752 (19.2)	778 (18.7)	

**Table A3:** Additive and multiplicative effects of NAc functional connectivity with the frontoparietal network on BMI at baseline based on sex.

	b	SE	*t*	p	sig
**Model 3**					
NAc functional connectivity with the frontoparietal network	0.77706	0.33632	2.31	0.020885	*
Sex (Male)	−0.29206	0.08307	−3.52	0.0004409	***
NAc functional connectivity with the frontoparietal network × Sex (Male)	−1.32806	0.44780	−2.97	0.0030286	**

Outcome: BMI. Control variables: age, race, ethnicity, parental education, household income, and family marital status. Model also controlled for the nested data. * *p* < 0.05; ***p* < 0.01; *** *p* < 0.001.
